# A locally advanced pancreatic body cancer presenting common bile duct invasion resected via distal pancreatectomy after gemcitabine plus nab-paclitaxel chemotherapy: A case report

**DOI:** 10.1016/j.ijscr.2022.106818

**Published:** 2022-02-07

**Authors:** Hiroshi Kawasaki, Mayumi Hoshikawa, Yusuke Kyoden, Tatsuo Iijima, Hiroshi Kojima, Junji Yamamoto

**Affiliations:** a Department of Gastrointestinal Surgery, Ibaraki Prefectural Central Hospital, 6528 Koibuchi, Kasama-city, Ibaraki 309-1793, Japan; b Department of Pathology, Ibaraki Prefectural Central Hospital, 6528 Koibuchi, Kasama-city, Ibaraki 309-1793, Japan; c Department of Oncology, Ibaraki Prefectural Central Hospital, 6528 Koibuchi, Kasama-city, Ibaraki 309-1793, Japan

**Keywords:** Case report, Organ-sparing surgery, Gemcitabine plus nab-paclitaxel chemotherapy, Pancreatic cancer, Distal pancreatectomy with celiac axis resection

## Abstract

**Introduction:**

The locally advanced pancreatic cancer has been steadily recognized as a potentially curable disease by a combination of chemotherapy and surgery. The remarkable effect of advanced chemotherapy would help surgeons do a function-preserving operation for advanced pancreatic cancer.

**Presentation of case:**

A 73-year-old woman presenting with obstructive jaundice was diagnosed to have a 3-cm pancreatic body cancer invading the celiac axis (CA), superior mesenteric artery (SMA), portal/splenic vein confluence, and the common bile duct (CBD). A plastic internal stent tube was placed endoscopically. After 11 cycles (231 days) of a weekly doublet chemotherapy with 1000 mg/m^2^ of gemcitabine and 125 mg/m^2^ of albumin-bound paclitaxel, the tumor shrunk based on imaging done every four months during chemotherapy, with residual periarterial high-density area around CA and proximal SMA and the patient was referred for surgery. During the operation, the absence of cancer cells was confirmed at (1) the origin of the proper hepatic artery, gastroduodenal artery and the left gastric artery, and (2) pancreatic cut stump along the right border of the portal vein; thus, distal pancreatectomy with coeliac axis resection was done. The patient had postoperative adjuvant chemotherapy with 100 mg/day of tegafur/gimeracil/oteracil for half a year and is currently alive and well, without signs of recurrence and diabetes mellitus a year after surgery.

**Discussion:**

Although surgical techniques aimed at local radicality are important, especially for conversion surgery for locally advanced pancreatic cancer, surgeons should consider the balance between radicality, safety, and functional preservation of surgery.

## Highlights

• A locally advanced pancreatic cancer has been curable by chemotherapy and surgery.
• The arterial invasion by imaging diagnostics is sometimes released by chemotherapy.
• The common bile duct invasion was dissolved after chemotherapy.
• The effect of chemotherapy would help surgeons do a function-preserving operation.

## 1 Introduction

The use of multiagent chemotherapy regimens, such as FOLFIRINOX and gemcitabine/albumin-bound paclitaxel (GnP), has been greatly advanced for the curative treatment of locally advanced pancreatic cancer (LAPC) [1], [2], [3]. LAPC has been increasingly recognized as a potentially curable disease by a combination of chemotherapy and surgery [4]. Herein, we present a case of a patient with LAPC involving the CA, SMA and common bile duct (CBD) at initial diagnosis and had a curative resection by distal pancreatectomy with CA resection (DP-CAR) [5], [6], preserving the pancreatic head after the outstanding effect of chemotherapy. This case report has been written following the SCARE 2020 criteria [7].

## 2 Presentation of case

A 73-year-old Japanese woman with a complaint of epigastric fullness and brown urine visited a medical practitioner. Due to abnormal liver enzyme levels based on blood chemistry, the patient was referred to our hospital. The patient was 146 cm in height and 49.8 kg in weight, and three months prior to visit to our hospital, she had had no body weight loss. She had no comorbidities, except for hyperlipidemia for over 13 years, and she never drunk nor smoked. The blood chemistry at initial hospital visit showed elevated levels of serum total bilirubin (3.9 mg/dL; normal range: 0.4–1.5 mg/dL), aspartate aminotransferase (378 U/L; normal range: 13–30 U/L), alanine aminotransferase (451 U/L; normal range: 7–23 U/L), and alkaline phosphatase (2156 U/L; normal range: 106–322 U/L). The fasting blood glucose of the patient was slightly elevated (116 mg/dL; normal range: 73–109 mg/dL), while the HbA1c level was normal (5.3%; normal range: 4.9–6.0%). The serum levels of pancreatic cancer tumor markers were all within normal range, as CA19–9 was 24.2 U/mL [<37], CEA was 2.3 ng/mL [<5.0], DUPAN-2 was 95 U/mL [≤150], and SPan-1 was 23.2 U/mL [≤30].

**Fig. 1:**
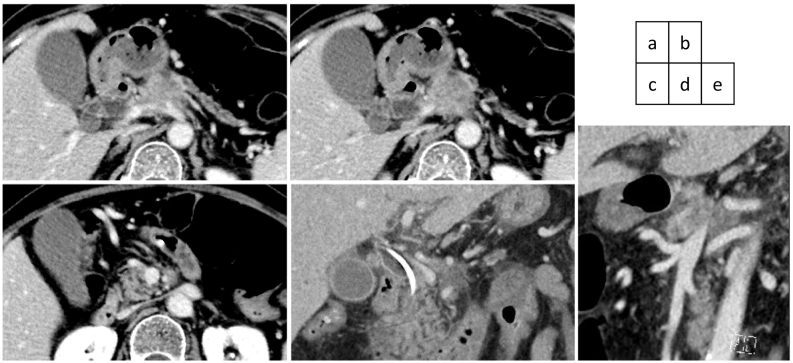
(a) The pancreatic body tumor invaded the celiac axis, splenic artery, and common hepatic artery. (b) The tumor also invaded the portal/splenic junction. (c) The tumor invaded more than 180 degrees of the superior mesenteric artery. (d) (e) The coronal (d) and sagittal (e) section images indicated the tumor invasion of the common hepatic artery, splenic artery, superior mesenteric artery, and portal vein.

**Fig. 2:**
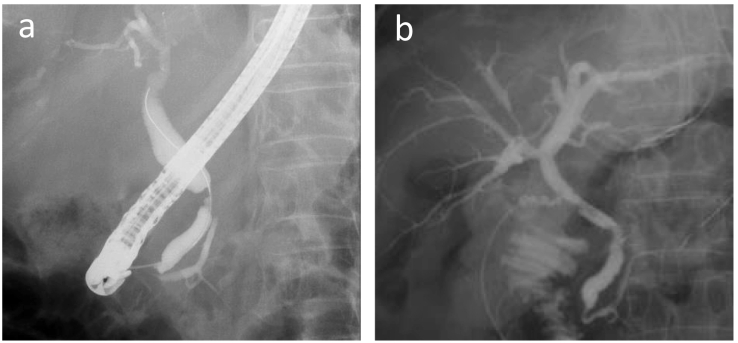
(a) Prior to preoperative chemotherapy, an endoscopic cholangiography showed common bile duct obstruction near the upper border of the pancreas. (b) The intraoperative cholangiography via the cystic duct revealed the relieved obstruction and ragged margin of the common bile duct.

An abdominal computed tomography (CT) scan with dynamic enhancement revealed a 3-cm, ill-defined tumor in the pancreatic body circumferentially invading the CA and SMA (Fig. 1a,c,d,e). The portal vein (PV) was narrowed, and the splenic vein was obliterated by the invading tumor (Fig. 1b,d,e). Nine days after the initial check-up, the serum bilirubin level was 9.5 mg/dL, and endoscopic biliary decompression was done. The CBD was obstructed near the upper border of the pancreatic head (Fig. 2a). A 7 Fr-thick, 7 cm-long plastic internal stent tube was placed. A gadoxetic acid-enhanced magnetic resonance imaging did not reveal any liver metastases. The patient was diagnosed to have a T4N0M0 clinical stage III pancreatic tumor.

**Fig. 3:**
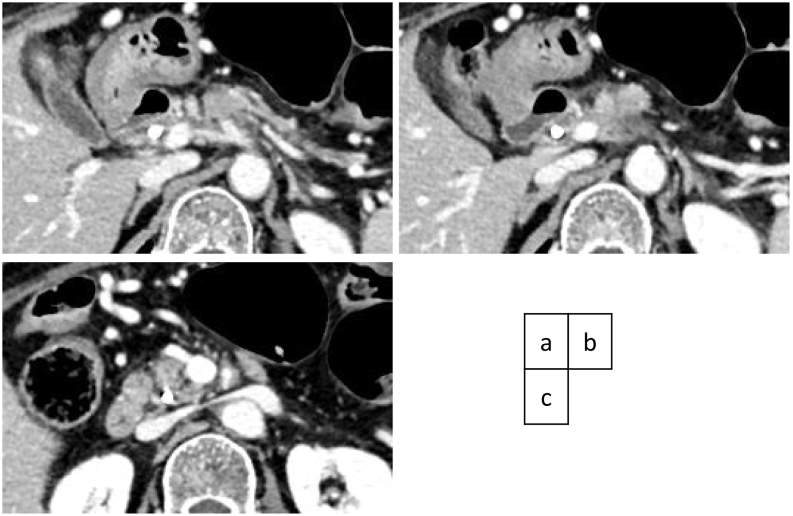
(a) (b) After 11 cycles of chemotherapy, the tumor contracted, and the portal vein was opened. (a) (c) The tumor shrunk, with residual periarterial high-density area around the celiac axis (CA) and proximal superior mesenteric artery (SMA).

A weekly doublet chemotherapy with 1000 mg/m^2^ of gemcitabine and 125 mg/m^2^ of albumin-bound paclitaxel for three to four weeks was started 40 days after the first visit to our hospital. Because the patient suffered from grade 4 neutropenia after the first two administrations during the first cycle of the doublet regimen, the dose was set at 70% of full dosage and was administered two to three weeks. The effect of chemotherapy was estimated by a monthly evaluation of tumor markers and CT scan every four months. The tumor shrunk on each imaging evaluation during chemotherapy, with residual periarterial high-density area around the CA and proximal SMA (Fig. 3a,b,c). After 11 cycles (231 days) of chemotherapy with 60% relative dose intensity, the patient was referred for surgery. As the edge of the shrunk tumor appeared to regress apart from the CBD, the internal stent tube was removed one month prior to surgery.

**Fig. 4:**
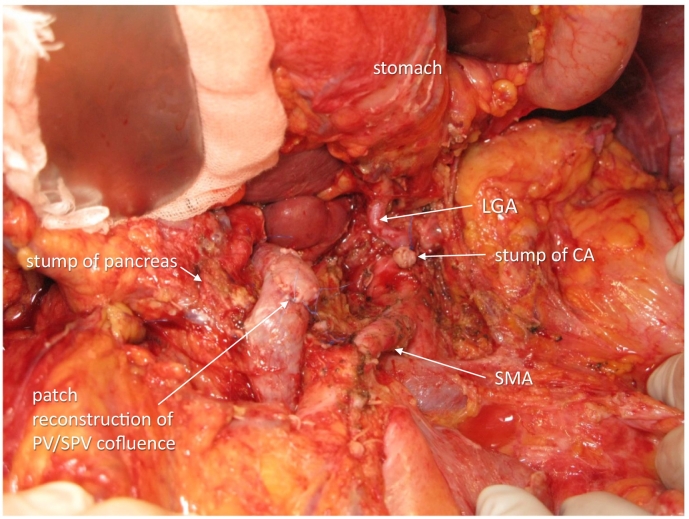
The view after resection. The stump of the CA was severed just distal to the left gastric artery (LGA). The surrounding perivascular tissues 4 cm proximal to the SMA was circumferentially dissected. The cut section of the pancreas was at the right of the portal vein. The widely resected portal vein/splenic vein (PV/SPV) confluence was reconstructed with right external iliac vein patch.

The operation was done 309 days after the first hospital visit and 29 days after the last administration of chemotherapy. The abdomen was entered via upper to middle midline skin incision. No liver and peritoneal metastases were observed. The absence of cancer cells in the nerve plexus was confirmed at (1) the origin of the proper hepatic artery and gastroduodenal artery and (2) the origin of the left gastric artery. The pancreas was cut along the right border of the PV, and there were no cancer cells with patchy deciduation of exocrine tissues and fibrous tissue proliferation at the cut surface; thus, DP-CAR, preserving the left gastric artery, was selected asss a preferable procedure [8], [9], [10]. The CA was severed just distal to the origin of the left gastric artery. The nerve plexus around the SMA was circumferentially dissected 4 cm from its origin. The confluence of the splenic vein to the SMV/PV was widely resected and reconstructed with the right iliac vein patch (Fig. 4). The operative time was 646 min, and blood loss was 873 g. Postoperatively, the patient suffered from slight anorexia but with no pancreatic fistula and was discharged on postoperative day 25.

The gross examination of the surgical specimen revealed 43 × 22 × 14-mm, ill-defined, pancreatic body tumor with an irregular whitish area and loss of lobulation. Microscopically, the tumor was a moderately differentiated tubular adenocarcinoma, encroaching beyond the pancreatic capsule but showing absence of invasion to the adventitia of resected CA and PV; thus, it was assigned as ypT3, ypN2 (four metastases out of 50 harvested nodes), ypM0, ypStage III according to the 8th edition of TNM classification. The cancer cells were observed very near to but more than 1 mm from the cut surface.

The patient had a postoperative adjuvant chemotherapy with 100 mg / day of S-1® (Tegafur/gimeracil/oteracil) for half a year and is currently alive and well, without signs of recurrence and diabetes mellitus a year after surgery.

## 3 Discussion

Owing to increasing safety with mortality rates under 5% [11], [12] during the last three decades, indications for pancreatic surgery have gradually extended to include those who demonstrate significant results after neoadjuvant therapy. Along with metastatic PDAC, LAPC, which was previously considered as an incurable disease and as an indication for palliative therapy, is now recognized as a potentially curable disease [13], [14]. According to a recent real-world study of an unselected cohort, 9% of patients with LAPC who underwent surgical resection after primary chemotherapy had favorable survival outcomes [15].

The expected effects of chemotherapy for LAPC prior to resection are (1) to provide a patient selection by testing the chemoresponsiveness of the tumor to identify aggressive diseases and to spare ineffective therapies and (2) to increase the complete resection rate by shrinking the tumor and treating micrometastases. As for surgical procedures after chemotherapy, almost all patients with LAPC with more than 180 degree of invasion to the CA and/or SMA spared combined resection of such major arteries [13]. An accompanying merit of marked tumor contraction is to facilitate a limited resection, which can omit the combined resection of surrounding tissues and maintain the postoperative quality of life. In the present patient, among three important structures (CA, SMA, and CBD), only CA was resected with the tumor because such resection alleviates the complete removal of the tumor without reconstructive procedures, and CA was diagnosed as the most extensively involved by the tumor arising from the pancreatic body. The CBD obstruction was resolved prior to resection. The pancreatic head was preserved after confirming the absence of tumor cells at the pancreatic cut stump along the right border of the PV. An intraoperative fluorography via the cystic duct revealed a well-lumened CBD with slightly ragged margin, reflecting fibrotic tissue proliferation after the regression of the tumor by chemotherapy. It is well-documented that a neoadjuvant treatment causes extensive pathological changes in the pancreas, resulting in a higher extent of fibrosis and pancreatic atrophy [16]. Pathology revealed the absence of invasion to the CA adventitia in the resected specimen. A collective study of 20 patients with DP-CAR reported such eliminating effect of chemotherapy [17].

Recently, FOLFIRINOX has been the major regimen for the neoadjuvant treatment of LAPC with high rate of conversion [3], [14], [15]. A reported series of successful conversion surgery includes relatively younger patients with age ranging from 56 to 66 years [3], [13], [14]. The present patient showed marked myelosuppression after the administration of two full doses of GnP. The patient had a remarkable response to chemotherapy with 60% relative dose intensity and less adverse effects. A prospective cohort study of unselected patients with borderline resectable or LAPC indicated a less frequent chemotherapy completion and conversion surgery in older patients (> 75 years) than in younger (≤ 75 years) patients [15]. A systemic therapy with less adverse effects is expected for year-after-year aging populations with pancreatic cancer.

Surgical procedures have been continuously refined and have markedly improved the survival outcomes of patients with pancreatic cancer, in combination with more effective adjuvant chemotherapy regimens [18]. Even the vessels diagnosed as circumferentially involved by the tumor can be preserved after an effective neoadjuvant treatment. The organ-sparing procedure for cancer includes merit and demerit for the patients. A merit would be preserving the relative dose intensity of adjuvant chemotherapy by maintaining nutrition and immune function by preserving bowel function. Some studies have reported that nutritional status, represented by serum albumin levels, and body weight and lean body mass is considered to affect adherence of patients with PC to postoperative chemotherapy and the reduced relative dose intensity of adjuvant chemotherapy has a negative impact on postoperative survival [19], [20], [21], [22]. Meanwhile, as a demerit, such conservative procedure always carries the risk of incomplete resection. Radiological assessment of invasive front of the tumor are no longer applicable after a neoadjuvant treatment [2], an appropriate use of intraoperative frozen section pathology is the key to achieve a complete resection.

## 4 Conclusions

Herein, we report a case of a patient with pancreatic body cancer who presented with obstructive jaundice and spared total pancreatectomy owing to the marked effects of preoperative chemotherapy. Although surgical techniques aimed at local radicality are important especially for the conversion surgery for LAPC, surgeons should progressively consider the balance between the radicality, safety, and functional preservation of surgeries.

## Ethics approval and consent to participate

Not applicable as per institutional policy.

## Consent for publication

Written informed consent was obtained from the patient for publication of this case report and accompanying images. A copy of the written consent is available for review by the Editor-in-Chief of this journal on request.

## Availability of data and materials

The data that support the findings of this study are available from the corresponding author, [JY], upon reasonable request.

## Sources of funding

All authors received no financial compensation related to the development of the manuscript.

## Research registration

N/A.

## Guarantor

Junji Yamamoto

## Provenance and peer review

Not commissioned, externally peer-reviewed.

## CRediT authorship contribution statement

HK drafted the manuscript and JY and HK participated in treating the patients and revised the manuscript. JY, MH, and YK participated in the surgery and postoperative management. TI investigated these cases pathologically. All authors read and approved the final manuscript.

## Declaration of competing interest

All authors have no affiliations with or involvement in any organization or entity with any financial interest, or non-financial interest in the subject matter or materials discussed in this manuscript.
